# Interpretable clinical decision support systems in high-risk pregnancy: a scoping review of models, methods, and implementation

**DOI:** 10.1186/s12884-025-08614-9

**Published:** 2026-01-06

**Authors:** Imad El Badisy, Bouchra Assarag, Zakaria Belrhiti

**Affiliations:** 1 AI and Data Science Service, Mohammed VI Center for Research and Innovation, Rabat, Morocco; 2 National School of Public Health, Rabat, Morocco; 3 Mohammed VI International School of Public Health Mohammed VI University of Sciences and Health, Casablanca, Morocco

**Keywords:** Clinical decision support system, High-risk pregnancy prevention, Interpretable machine learning, Rule-based systems, Predictive modeling

## Abstract

Clinical Decision Support Systems (CDSS) powered by machine learning (ML) are increasingly recognized as valuable tools for improving maternal healthcare, particularly in the prevention of high-risk pregnancies. However, their adoption in real-world settings remains limited due to concerns about transparency, reproducibility, and integration into clinical workflows. Interpretable ML methods offer a promising solution by enhancing the usability and trustworthiness of these systems. This scoping review maps interpretable CDSS for maternal high-risk pregnancy prevention and intrapartum management, including both ML and rule-based systems. We examined model characteristics, implementation and validation approaches, and interpretability methods. We searched PubMed and supplemented results with targeted screening in Google Scholar. Included studies reported interpretable outputs and clinical performance. Key data extracted encompassed study design, CDSS type, validation strategies, interpretability techniques, and clinical outcomes. Nineteen studies met the inclusion criteria. Most ML studies used Random Forests or Support Vector Machines. non-ML systems commonly implemented standardized rules, scoring systems with early-warning alerts. Post hoc methods such as SHAP and LIME were frequently used. Reporting of code/data availability was variably documented, which may limited reproducibility. Most evaluations were retrospective, constraining generalizability. Future work should prioritize transparent, prospective, and open science practices, with interpretable outputs aligned to clinical reasoning for successful integration.

## Introduction

Maternal healthcare continues to face significant global challenges, particularly in regions with limited access to high-quality medical services. In recent years, CDSS have emerged as essential tools to assist healthcare professionals by integrating real-time patient data and providing evidence-based recommendations during critical moments in maternal care. With the rapid advancement of artificial intelligence (AI) and ML, the use of CDSS in maternity care has expanded significantly. These technologies hold considerable promise for improving maternal and neonatal outcomes by anticipating complications and optimizing clinical interventions [1, 2].

CDSS have shown potential across various aspects of maternity care, including risk stratification, early detection of complications, and decision-making support during labor and delivery [1–4]. For example, tree-based models such as Random Forests, kernel-based methods like Support Vector Machines (SVM), and deep learning models including neural networks, have been successfully applied to predict conditions like preeclampsia and preterm birth, which facilitate earlier and more targeted interventions [2]. Despite these advances, significant challenges remain, particularly concerning model interpretability, data quality, and generalizability across diverse populations, which is essential in maternal health due to substantial variation in demographics, socioeconomic status, and healthcare settings [2, 5, 6].

Several systematic reviews have examined the clinical impact of CDSS, often reporting favorable outcomes such as reductions in maternal and neonatal morbidity. However, substantial variability exists in how these systems are designed, implemented, and evaluated, making it difficult to synthesize evidence and draw generalizable conclusions [1]. To explore this gap, a scoping review is warranted to map existing approaches, explore current methodologies, and identify key limitations within the topic [2, 6].

Given this variability and the persistent challenges in model interpretability, this scoping review intentionally includes both ML-based and non-ML (rule-based, heuristic, or surveillance) CDSS, provided they contribute to interpretable, data-driven decision support in maternal or perinatal care. We examine validation strategies and performance metrics, evaluate associated clinical outcomes, and highlight existing research gaps and future development opportunities.

## Methods

This scoping review comprehensively identifies and synthesizes the literature on interpretable CDSS for high-risk pregnancy prevention and intrapartum management.

### Eligibility criteria

Studies were eligible if they described CDSS developed for high-risk pregnancy prevention or intrapartum management, using ML or non-ML approaches, and provided details on interpretability, clinical performance, and, where applicable, implementation. Peer-reviewed journal articles and preprints published in English between January 2012 and August 2023 were considered. We excluded studies that lacked explicit information on CDSS implementation, interpretability methods, or relevant clinical outcomes, as well as studies published in languages other than English.

#### Search strategy

**Fig. 1:**
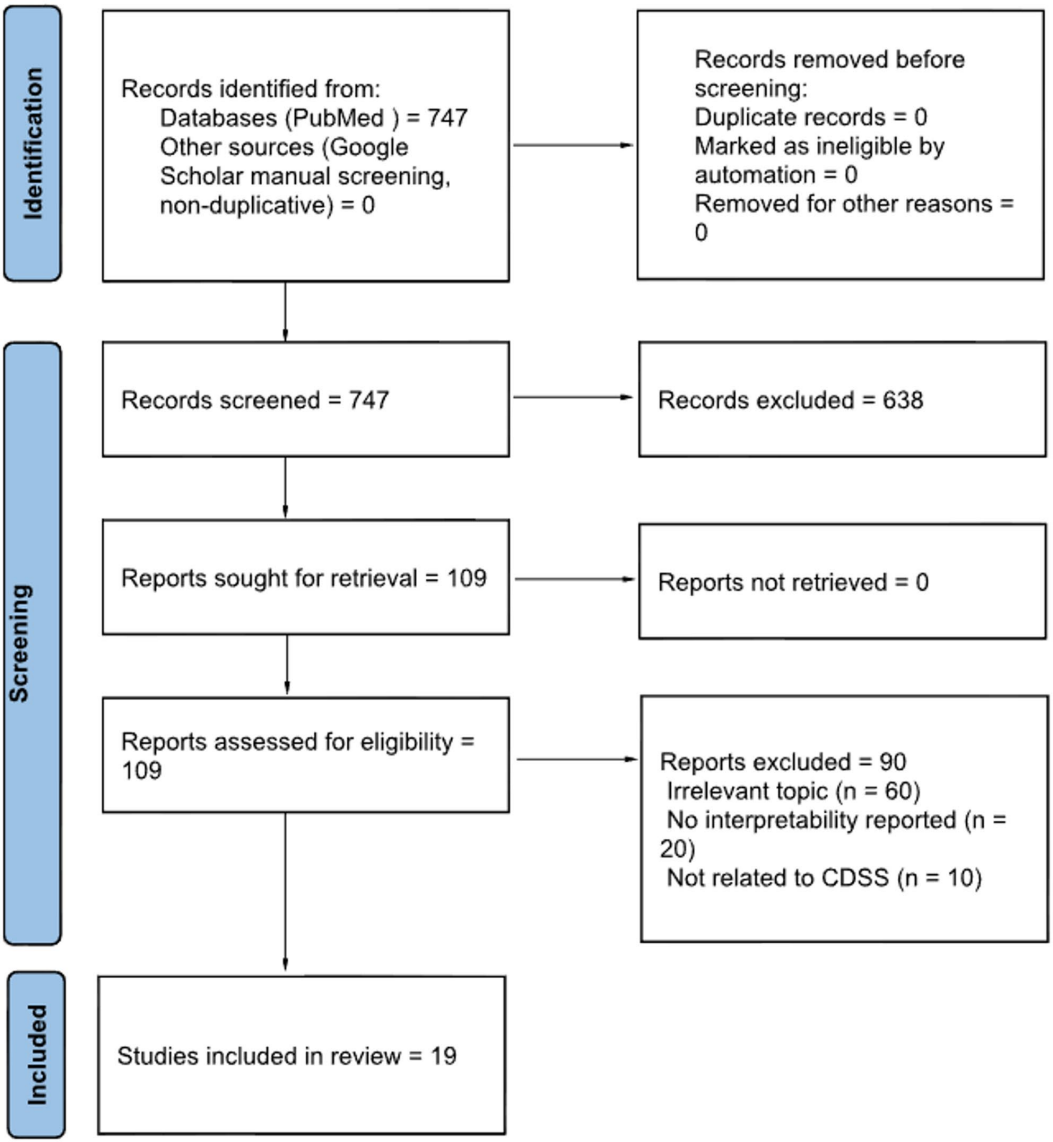
PRISMA 2020 flow diagram illustrating the study selection process, from identification to inclusion of the final 19 studies

We conducted a systematic search in PubMed as the primary database. We then supplemented this search with targeted screening in Google Scholar to improve coverage of CDSS and informatics-oriented publications. Records retrieved from Google Scholar were merged with PubMed results and deduplicated prior to screening, consistent with the counts reported in the PRISMA flow diagram (Fig. 1).

The search strategy incorporated extensive keywords related to interpretability, explainable artificial intelligence (XAI), deep learning, and CDSS, with a particular emphasis on applications in maternal health and high-risk pregnancy prevention. Terms specifically related to pregnancy complications, were also included to ensure optimal coverage.

The strategy was structured into five thematic groups, each addressing specific aspects of CDSS (Table 1). Natural language processing and text-mining techniques were applied to refine queries and enhance retrieval precision.

**Table 1:** Search strategy structure used to identify relevant studies on interpretable ML in CDSS for high-risk pregnancy prevention

Search topic	Search terms	AND	Additional terms
Topic 1	“interpretable machine learning” OR “explainable AI” OR “explainable artificial intelligence” OR “XAI”	AND	“clinical decision support system” OR “CDSS” OR “decision support” AND “high-risk pregnancy prevention” OR “pregnancy” OR “gestational diabetes” OR “maternal health”
Topic 2	“deep learning” OR “DL” OR “neural networks” OR “CNN” OR “RNN” OR “LSTM”	AND	“clinical decision support system” OR “CDSS” OR “decision support” AND “high-risk pregnancy prevention” OR “pregnancy” OR “gestational diabetes” OR “maternal health”
Topic 3	“machine learning” OR “ML” OR “supervised learning” OR “unsupervised learning” OR “reinforcement learning”	AND	“interpretability” OR “transparency” OR “explainability” AND same as above
Topic 4	“performance metrics” OR “outcomes” OR “accuracy” OR “sensitivity” OR “specificity” OR “AUC-ROC”	AND	“interpretability” OR “transparency” OR “explainability” AND same as above
Topic 5	“implementation” OR “validation” OR “clinical setting” OR “integration” OR “user interface” OR “usability”	AND	“interpretability” OR “transparency” OR “explainability” AND same as above

### Study selection

Titles and abstracts were initially reviewed based on the inclusion criteria. Potentially eligible studies were then evaluated in detail through full-text assessments. Screening of titles/abstracts and full texts was conducted by one reviewer, with methodological oversight and verification by a second reviewer. Uncertainties were resolved by discussion. Figure 1 presents the PRISMA 2020 flow.

### Data extraction

Extracted details included publication metadata (title, authors, year, source), study design (retrospective, prospective), sample size, and population characteristics. We also documented model types, main features included, training and validation strategies, and implementation contexts. Interpretability techniques employed, clinical integration settings, and system usability features were specifically noted. We also captured performance metrics, such as accuracy, sensitivity, specificity, and AUC-ROC, and clinical outcomes like reduced adverse events or improved diagnostic accuracy. Where available, comparisons with traditional approaches, healthcare professional feedback, and reported challenges with recommendations for improvement were also recorded.

### Data synthesis

Data were synthesized using a combination of descriptive statistics and thematic analysis. Numerical summaries were used to report the frequency and distribution of study characteristics, model types, and performance metrics, while thematic analysis identified common challenges and patterns related to interpretability, clinical integration, and implementation. Results are organized around the main research objectives, focusing on ML model types, validation strategies, interpretability techniques, and clinical outcomes. Findings are presented through narrative synthesis and are supported by visual summaries, including tables and figures that illustrate key trends and implementation patterns.

## Results

Table 2 summarizes the characteristics of the 19 included studies, highlighting their geographic origin, publication details, and the clinical outcomes targeted by each CDSS intervention. The studies reviewed span a range of research articles published between 2012 and 2023. They originate from various countries, including the United Kingdom [7], the United States [8], Portugal [9], Spain [10], Italy [11], Jordan and Australia [12], India [13], Kenya [14], and Tanzania [15].

**Table 2:** Overview of the 19 included studies, including reference details, country of origin, and the clinical outcome addressed

ID	References	Title	Country	Clinical outcome targeted
1	Fergus, Selvaraj, and Chalmers (2018) [3]	Machine learning ensemble modelling to classify caesarean section and vaginal delivery types using Cardiotocography traces	United Kingdom	Assisted clinical decision-making to classify cesarean section and normal vaginal deliveries.
2	Hutchinson-Colas et al. (2023) [16]	New Jersey maternal mortality dashboard: an interactive social-determinants-of-health tool	United States	Informed policymakers and healthcare professionals about maternal mortality trends.
3	Sufriyana, Wu, and Su (2021) [17]	Human and machine learning of prognostic prediction for prelabor rupture of membranes and the time of delivery	Taiwan and Indonesia	Predicted time of delivery for patients with prelabor rupture of membranes (PROM).
4	Yang et al. (2022) [18]	Deep learning-based prognosis prediction among preeclamptic pregnancies using electronic health record data	United States	Predicted time to delivery among preeclamptic pregnancies based on EHR data.
5	Eberhard et al. (2023) [19]	An interpretable longitudinal preeclampsia risk prediction using machine learning	United States	Identified preeclampsia risk and at-risk patients earlier than current clinical practices.
6	Comer et al. (2020) [20]	The MITRE Maternal Mortality Interactive Dashboard (3MID)	United States	Evaluated the effect of quality improvement toolkits in reducing maternal mortality.
7	Rawashdeh et al. (2020) [12]	Intelligent system based on data mining techniques for prediction of preterm birth for women with cervical cerclage	Jordan, Australia	Predicted preterm birth risk and timing of spontaneous delivery after cerclage.
8	Georgieva, Redman, and Papageorghiou (2017) [7]	Computerized data-driven interpretation of the intrapartum cardiotocogram: A cohort study	United Kingdom	Optimized detection of fetal distress and reduced unnecessary operative deliveries.
9	Uccella et al. (2012) [11]	Prediction of fetal base excess values at birth using an algorithm to interpret fetal heart rate tracings	Italy	Predicted fetal acidemia at birth based on fetal heart rate monitoring.
10	Fernandez, Fernández, and Sanchez (2019) [10]	A decision support system for predicting the treatment of ectopic pregnancies	Spain	Improved treatment decision accuracy, especially in avoiding unnecessary surgeries.
11	Bartlett et al. (2021) [14]	Design, development, and implementation of a novel digital health tool for skilled birth attendants	Kenya	Improved decision-making during labor and delivery, reduced maternal mortality, and enhanced quality of care.
12	Durand et al. (2012) [21]	Design and usability of heuristic-based deliberation tools for women facing amniocentesis	United Kingdom	Improved decision-making processes for women considering amniocentesis.
13	Lopes-Pereira et al. (2019) [9]	Computerized analysis of cardiotocograms and ST signals is associated with significant reductions in hypoxic-ischemic encephalopathy and cesarean delivery	Portugal	Reduced hypoxic-ischemic encephalopathy (HIE) and unnecessary cesarean deliveries.
14	Saha (2021) [13]	Decision support system incorporating data mining for maternal and child health system strengthening	India	Supported intervention planning and improved maternal and child health outcomes.
15	Klumpner et al. (2018) [22]	Use of a novel electronic maternal surveillance system to generate automated alerts in labor and delivery	United States	Reduced delays in detecting maternal morbidity by providing timely alerts.
16	Patterson et al. (2023) [23]	Predictive model of low birth weight in low- and middle-income countries: A prospective cohort study	Multi-country (8 LMIC sites)	Identified predictors of low birth weight across diverse maternal populations.
17	Kovacs et al. (2021) [15]	Developing practical tools for predicting neonatal mortality at a neonatal intensive care unit	Tanzania	Predicted neonatal mortality using vital signs and clinical risk factors.
18	Sitek et al. (2023) [24]	Artificial intelligence in the diagnosis of necrotizing enterocolitis in newborns	Poland, United States	Improved early diagnosis of NEC to reduce mortality and severe complications.
19	Ravindra et al. (2023) [8]	Deep representation learning for associations between physical activity and prematurity	United States	Identified links between sleep/activity patterns during pregnancy and risk of preterm birth.

The included studies addressed a range of clinical outcomes, including cesarean section classification [3], maternal mortality [20], preterm birth [12], neonatal mortality [15], and decision-making for ectopic pregnancy treatment [10]. While some focused on specific complications such as preeclampsia [19] or fetal acidemia [11], others examined broader population-level tools, such as the New Jersey maternal mortality dashboard [16] and the MITRE Maternal Mortality Interactive Dashboard [20].

Methodologies varied considerably. Several studies used advanced ML models, such as deep learning-based prognostic tools for preeclampsia [18], while others employed rule-based or data-driven approaches, such as Omniview-SisPorto for cardiotocography analysis [9]. Some studies prioritized implementation in real-world clinical workflows, like the iDeliver platform used by skilled birth attendants in Kenya [14].

Reported outcomes included improved decision-making support [14], better prediction of adverse events [11], and more effective maternal health management [9]. However, the variation in model types, clinical settings, and targeted outcomes highlights the heterogeneity of CDSS applications in maternal care.

### Types of ML models used

**Fig. 2:**
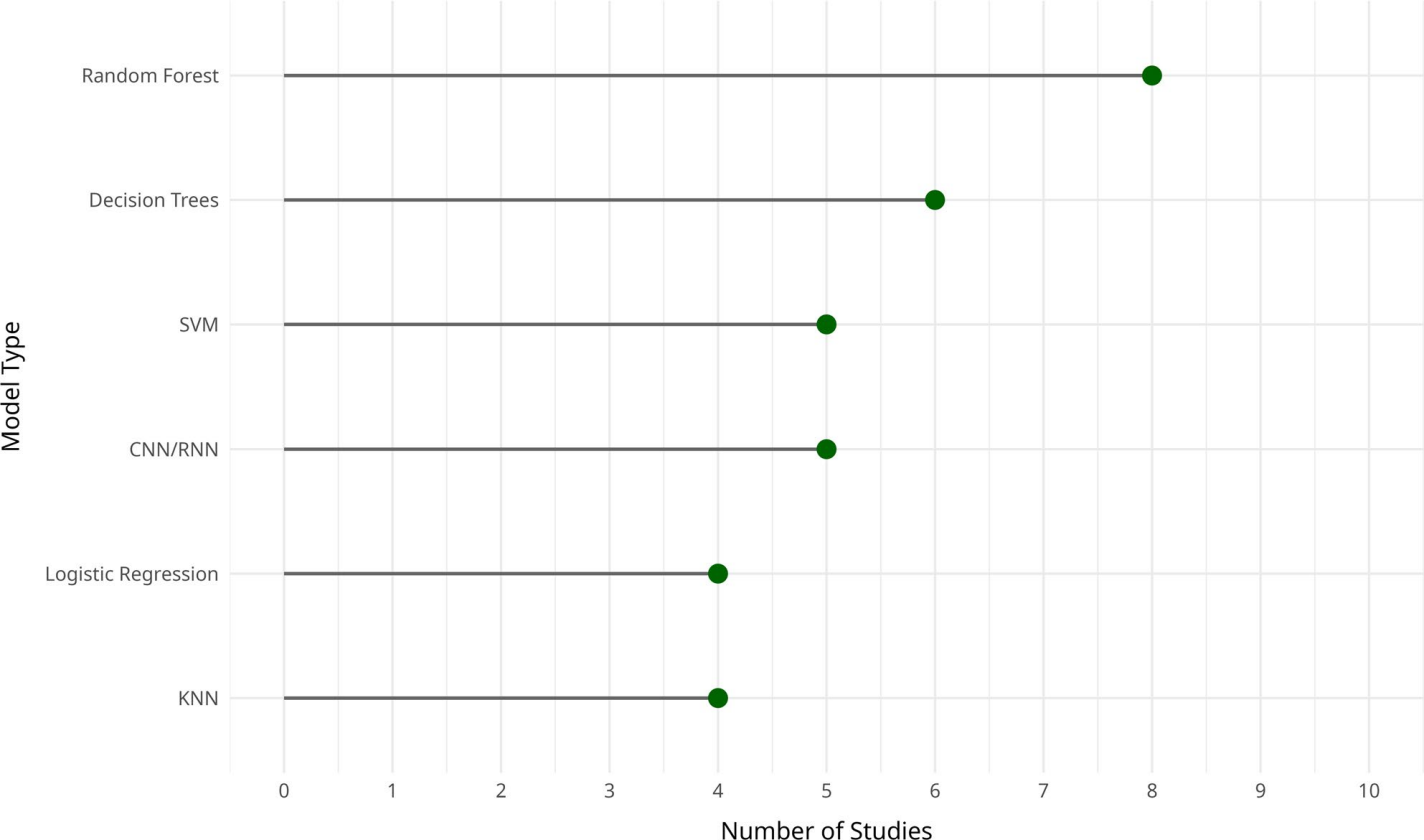
Frequency of ML and deep learning models used in CDSS for high-risk pregnancy prevention

The ML models employed across the included studies varied widely (Fig. 2). Commonly used models included Fisher’s Linear Discriminant Analysis (FLDA), Random Forest (RF), and Support Vector Machines (SVM), which were frequently applied for predictive modeling in clinical contexts [3]. Some studies focused on exploratory analysis without using formal ML algorithms, instead relying on dashboards and simulation tools to visualize trends and inform decision-making [16].

Several studies implemented more advanced or customized models. These included deep-insight visible neural networks (DI-VNN), Ridge Regression (causal RR), Elastic Net Regression (PC-ENR), and Gradient Boosting Machines (PC-GBM), particularly for complex prognostic tasks [17]. In the context of survival analysis, Cox-nnet v2 and the traditional Cox Proportional Hazards model were used to predict time-to-event outcomes in high-risk pregnancies [18]. Other studies adopted ensemble or hybrid approaches, combining models such as linear regression, Random Forest, XGBoost, and deep neural networks to build multi-output prediction frameworks [19].

In a few cases, the studies did not involve predictive models per se but rather simulations based on demographic variables and maternal mortality indicators to support policy development and strategic planning [20]. A broader array of models, including decision trees, k-nearest neighbors (KNN), artificial neural networks (ANN), Gaussian processes, K-star, and locally weighted learning (LWL), was used to address different facets of high-risk pregnancy prediction and classification [12].

Some studies focused on real-time monitoring and integrated risk assessment. For example, the OxSys 1.5 system combined clinical indicators with cardiotocography (CTG) data to support intrapartum decision-making [7]. Rule-based approaches were also used, particularly in systems designed to interpret fetal heart rate patterns using standardized clinical rules and heuristics [11].

More sophisticated architectures such as multilayer perceptrons (MLP), convolutional neural networks (CNN), and deep learning frameworks were applied in studies aiming to enhance prediction of pregnancy-related complications [10]. Other research prioritized the development of digital tools and interfaces without explicit ML algorithms, instead focusing on structuring clinical decision-making and improving care delivery [14].

Several studies applied advanced mathematical models to analyze physiological signals, such as uterine contractions, fetal heart rate variability, accelerations, and decelerations [9]. Others employed association rule mining, clustering, and visual analytics to extract patterns from routine clinical data and support broader maternal and child health system planning [13].

Predefined clinical algorithms, including those based on the Maternal Early Warning Criteria (MEWC), were used to detect abnormal vital signs and prompt timely responses to clinical deterioration [22]. Classical techniques such as decision trees, logistic regression, KNN, and SVM were combined with grid-search cross-validation to optimize model performance [23]. Generalized Linear Models (GLMs) and decision trees were applied for predicting neonatal mortality [15].

Finally, a range of classification and diagnostic models, including SVM, decision trees, Naive Bayes, Random Forest, and CNN, were used for identifying and assessing complex neonatal conditions like necrotizing enterocolitis [24]. Advanced deep learning models such as series2signal, CNN, KNN, and TimeSeriesForest were also applied to wearable sensor data to detect associations between physical activity, sleep patterns, and preterm birth risk [8].

### Validation techniques and performance

The reviewed studies employed a wide range of validation techniques to assess model performance, including holdout methods, cross-validation, and external validation using independent datasets. For example, Study 1 used a standard holdout split (80/20) along with 5-fold cross-validation, reporting strong performance metrics with 87% sensitivity, 90% specificity, and an AUC of 0.96 [3]. In contrast, Study 2 focused on exploratory analysis and did not report any formal validation approach [16].

Studies such as 3 and 4 adopted both internal and external validation strategies to ensure generalizability across settings, yielding C-index values between 0.71 and 0.75 for delivery time prediction [18]. Study 5 used an independent validation cohort from two hospitals and reported AUC values ranging from 0.73 to 0.91 at different prediction time points [19].

Some studies applied techniques like SMOTE to address class imbalance [12], while others, such as Study 6, did not report validation strategies, instead presenting simulation-based results [20]. Studies 9 and 10 used 10-fold cross-validation and achieved high classification accuracy (up to 96%) using SVM [10]. Study 8 relied on retrospective cohort analysis, reporting sensitivity and false-positive rates as key performance indicators [7], whereas Study 11 focused on usability testing rather than quantitative validation [14].

**Fig. 3:**
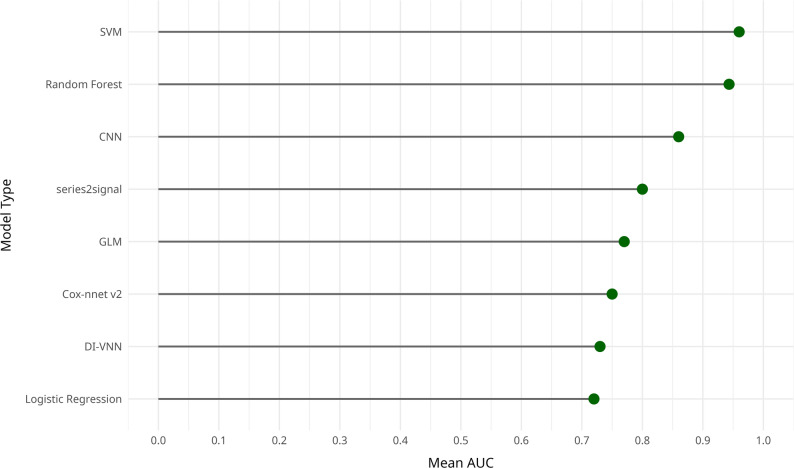
Average predictive performance (mean AUC) of ML and deep learning models used in CDSS for high-risk pregnancy prevention

Reported performance metrics varied, often depending on model type and study setting. For instance, Study 13 highlighted clinical impact, such as a 58% reduction in hypoxic-ischemic encephalopathy (HIE) and a 7% increase in instrumental vaginal deliveries [9]. Other studies, such as 16 and 17, reported standard metrics like AUC, precision, and recall [15], reflecting varied approaches to evaluating effectiveness (Fig. 3).

### Targets, predictors, and frameworks

The studies included in this review utilized a wide array of CDSS frameworks, each designed to address different aspects of maternal healthcare (Table 3). These ranged from systems focused on predicting specific conditions, such as preeclampsia, cesarean section, and timing of spontaneous delivery, to broader platforms for maternal health monitoring and decision-making.

**Table 3:** Overview of CDSS targets, predictors, and software frameworks used in the included studies

ID	Target	Predictors/Features	Framework or Software Used
1	Classify caesarean section and vaginal delivery types based on CTG signals.	13 features initially extracted, reduced to 8 after feature selection (DFA, RMS, FD, etc.)	R (RStudio); packages: Signal, MASS, hmeasure, pROC, ROCR, randomForest, caret, e1071, DMwR
2	Identifies trends and correlations between maternal mortality and social, demographic, and health factors.	Maternal mortality by age, race/ethnicity, insurance coverage, poverty rates, Medicaid coverage, obstetricians/midwives per capita.	R; Shiny web application framework
3	Prediction of PROM and estimation of time of delivery.	Medical history data, including ICD-10 codes for diagnoses and procedures.	R & Python; tools: RStudio, caret, keras, tensorflow
4	Prediction of time to delivery for preeclampsia.	Features included gestational age, severity of PE, maternal age, parity, gravidity, blood pressure, white blood cell count, platelet count, AST, etc.	Python 2.7 & R 4.0.2; R packages: mice, survival
5	Preeclampsia prediction using medical history, vital signs, and lab results.	Vital signs (systolic and diastolic blood pressure), interpregnancy interval, chronic hypertension, etc.	Python; packages: SHAP (v0.41.0), xgboost
6	Simulated reduction in maternal mortality rates based on toolkit implementation.	Demographic data (age, race, county of residence) as predictors for maternal mortality risk.	R; Shiny for interactive simulations and visualizations
7	Predicting pregnancy continuation and timing of spontaneous delivery post-cerclage.	Maternal age, obstetric history, cervical characteristics, gestational age, number of fetuses, progesterone use, symptoms of labor, type of cerclage.	WEKA (Java-based machine learning software)
8	Detecting fetal compromise and comparing clinical decision-making.	Non-classical cardiotocography features, decelerative capacity (DC), preeclampsia, thick meconium.	OxSys 1.5 (custom clinical prototype for CTG analysis)
9	Prediction of umbilical artery base excess at birth.	Fetal heart rate patterns, gestational age, and mode of delivery.	Standardized algorithm; no specific framework reported
10	Treatment decision support (Surgery, Medical Treatment, or Expectant Management).	Age, G, P, C, A, E, IVE, Location, Ectopic mm1, Ectopic mm2, Free Liquid, Initial beta-HCG levels.	RapidMiner Studio (graphical ML platform)
11	Enhanced record-keeping and decision support for labor management.	Maternal and fetal vital signs, labor progression, and acuity levels.	OpenMRS & CliniPAK (open-source EMR & health info systems)
12	Decision aids for helping women make informed decisions about amniocentesis.	Not applicable (user interaction with decision aids rather than predictive modeling).	Custom heuristic decision aid (non-ML)
13	Reduced HIE rates and cesarean deliveries, increased instrumental vaginal deliveries.	Uterine contractions, fetal heart rate variability, accelerations, decelerations, and ST signals.	Omniview-SisPorto (proprietary CTG/ST interpretation system)
14	Systemic knowledge generation for reducing healthcare inequities.	MCH indicators, educational, social, and economic parameters, healthcare interventions, and health system building blocks.	R; Shiny package
15	Alerts for abnormal vital signs.	Vital signs such as blood pressure, heart rate, pulse oximetry, and lab data.	AlertWatch™ OB (web-based clinical surveillance system)
16	Identification of maternal and pregnancy characteristics predicting low birth weight (LBW).	Maternal age, education, parity, height, weight, socio-economic status (SES), antenatal care visits, hypertensive disorders, severe antepartum hemorrhage, severe infection.	R; packages: rpart, ROCR
17	Neonatal mortality prediction.	Birthweight, heart rate, temperature, oxygen saturation, asphyxia.	R; packages: rpart, ROCR
18	AI-based early detection of necrotizing enterocolitis (NEC).	Abdominal ultrasound findings, pneumatosis intestinalis, bowel ischaemia, and microbiota data.	Multiple ML techniques (classical & deep learning); unspecified implementation
19	Gestational age prediction and detection of preterm birth risk.	Physical activity data (sleep and wake patterns), gestational age, self-reported clinical data (comorbidities like hypertension, diabetes).	Custom DL framework (series2signal)

The models employed a variety of predictors, including clinical indicators, demographic variables, and biochemical parameters. Common features included gestational age, maternal age, parity, prior medical history, and fetal heart rate patterns, highlighting the multidimensional nature of maternal risk assessment.

In terms of implementation, several programming languages and tools were used, with R and Python being the most prevalent. Many studies relied on R packages for model training and evaluation in web-based environments [19]. Others used custom-built platforms or proprietary systems, like OxSys 1.5 and Omniview-SisPorto, which were designed for real-time CTG monitoring and fetal distress prediction [9].

Open-source frameworks also featured prominently. Tools such as OpenMRS, Shiny dashboards, and other custom web applications were used to facilitate data collection, visualization, and interactive decision support [13]. These tools reflect a growing emphasis on accessibility, transparency, and real-time integration into clinical workflows.

### Interpretability methods

**Fig. 4:**
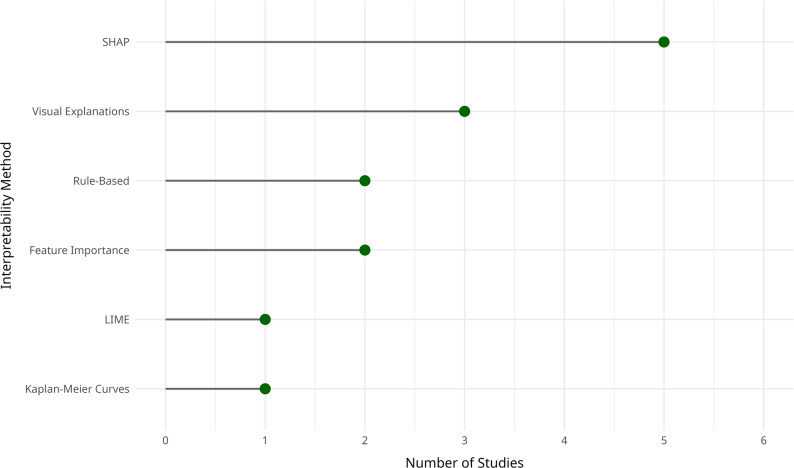
Frequency of interpretability methods used in CDSS for high-risk pregnancy prevention

The interpretability techniques reported across the reviewed studies varied widely (Fig. 4). Among these, SHAP (Shapley Additive Explanations) emerged as the most commonly used method for feature attribution. Multiple studies applied SHAP to highlight how individual variables contributed to the model’s output, particularly for predictions related to preeclampsia, neonatal complications, and gestational age [3, 8, 10, 17, 19].

Other studies employed visual interpretability techniques, such as Kaplan-Meier curves and Cox proportional hazards coefficients, particularly in the context of survival analysis and time-to-event predictions [18]. Local methods like LIME (Local Interpretable Model-Agnostic Explanations) were also used in a few cases to offer case-specific insights into individual predictions [17].

Some studies implemented rule-based or heuristic approaches, embedding clinical logic directly into the CDSS. These systems made use of pre-defined decision rules or scoring systems that were more intuitive for clinicians [11, 21].

Notably, several studies did not report any interpretability method [14, 16, 20, 23]. In these cases, the focus was often on system design, usability testing, or exploratory data analysis rather than on understanding how models reached their predictions. This absence of interpretability may limit clinical adoption, as transparency is a critical factor in building trust and ensuring safe integration of AI tools into decision-making processes.

Overall, these findings highlight the inconsistent use of interpretability techniques in the field and underscore the need for more structured, clinically relevant approaches. Consistently incorporating interpretable outputs, especially in high-stakes domains like maternal health, is essential to promote clinician engagement, trust, and ultimately, the effective use of CDSS.

### Clinical outcomes

**Fig. 5:**
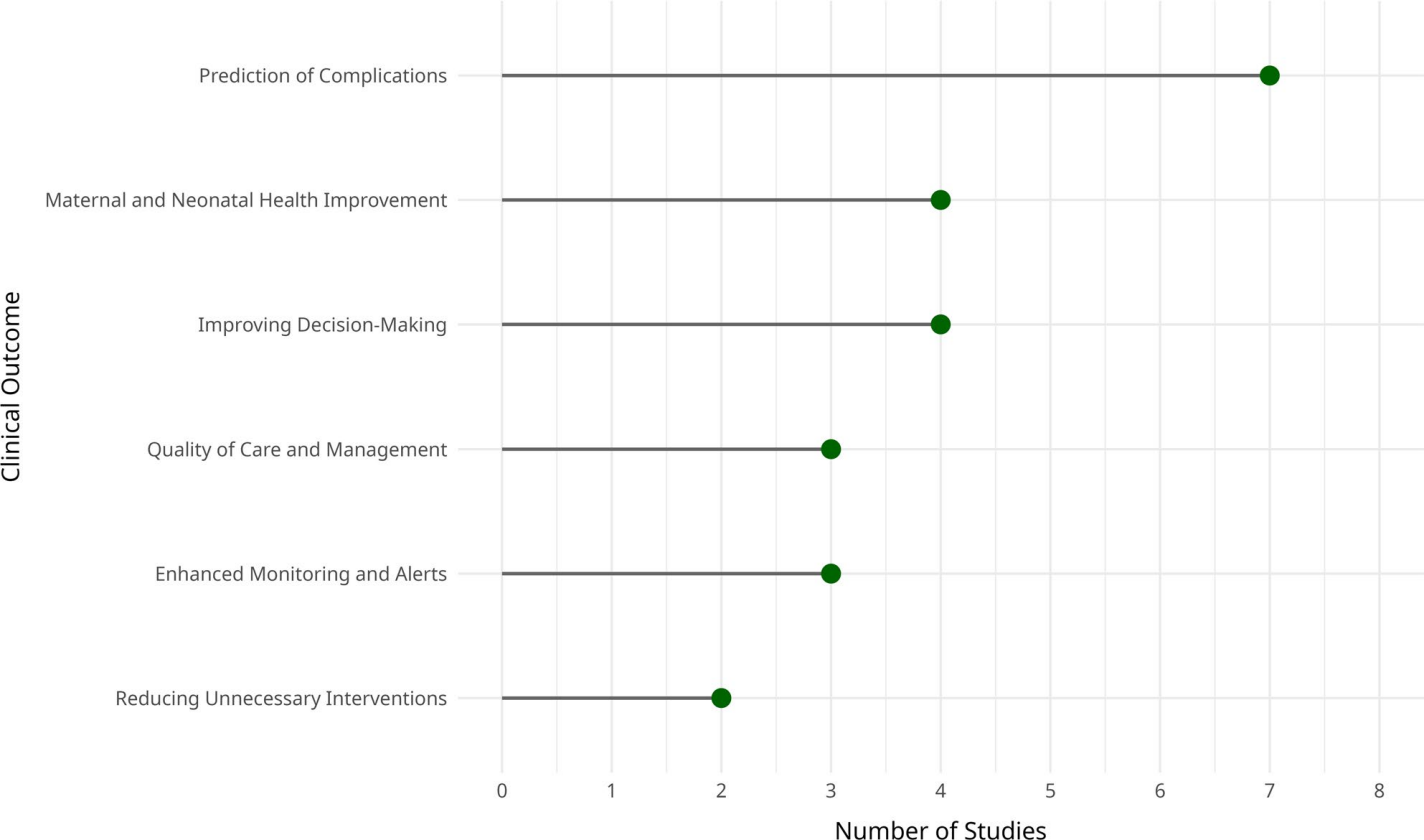
Distribution of clinical outcomes reported by CDSS models in high-risk pregnancy prevention

The reviewed studies reported a broad spectrum of clinical outcomes, with a central focus on improving maternal and neonatal health indicators (Fig. 5). Key findings included enhanced clinical decision-making for procedures such as cesarean section [3], as well as improved risk estimation for conditions like preeclampsia and preterm birth [18, 19]. These improvements enabled earlier and more tailored interventions, potentially reducing adverse outcomes.

Several studies also demonstrated effective CDSS applications in managing critical complications, such as fetal acidemia [11] and neonatal mortality [23]. Beyond individual-level outcomes, some systems contributed to broader health system impacts, notably reductions in maternal mortality [16, 20], and enhanced workflow efficiency that minimized delays in clinical response [22].

While many studies prioritized model performance, those centered on implementation and usability highlighted the importance of embedding CDSS tools within clinical routines. These studies stressed that real-world effectiveness depends, beyond the predictive accuracy, on user trust, seamless integration into care pathways, and actionable insights for healthcare professionals.

### Health professional involvement and suggested improvements

The extent of health professional involvement in the development and testing of CDSS varied widely across the reviewed studies. In several cases [3, 12, 18, 20], clinicians were not explicitly involved in system testing, raising concerns about the clinical relevance and real-world integration of these tools. Many of these studies prioritized algorithm development and retrospective validation, often overlooking direct user evaluation during early phases [19].

In contrast, some studies actively engaged healthcare providers throughout development. For instance, the iDeliver tool in Kenya incorporated iterative feedback from birth attendants to improve usability and ensure the tool aligned with local clinical workflows [14].

A number of studies also acknowledged the absence of prospective clinical validation as a significant limitation. For example, while the DI-VNN system for preeclampsia prediction was tested on both internal and external datasets, it lacked prospective application in hospital environments, limiting its generalizability [17]. Similarly, the New Jersey maternal mortality dashboard [16], developed with input from clinicians, policymakers, and researchers, would benefit from implementation in diverse geographic settings to further explore its effectiveness in tracking maternal health trends. The OxSys system [7], designed to detect fetal distress, also required further prospective validation to fine-tune alarm thresholds and minimize false positives before broader clinical deployment.

In several cases, healthcare professionals directly contributed to system evaluation. For instance, obstetric consultants independently assessed fetal heart rate tracings in a study on fetal monitoring, providing feedback that helped reduce both false-positive and false-negative rates [11]. Likewise, a CDSS implemented over 14 years in a tertiary care hospital [9] benefitted from continual clinician feedback, demonstrating the value of long-term real-world implementation studies in refining these systems.

Common suggestions for improving CDSS centered on enhancing generalizability and expanding training datasets. Many studies emphasized the need for multi-center validation to ensure broader applicability across healthcare settings [8, 19, 24]. For example, the AI-based CDSS designed for NICU use in Tanzania [15] would gain from incorporating data from a wider variety of hospitals to boost reliability and contextual fit. Similarly, the series2signal model for predicting gestational age and preterm birth was flagged for further validation through randomized controlled trials to confirm causality and robustness [8].

For tools focused more on usability than on prediction accuracy, such as the decision aids for amniocentesis [21], user feedback served as the main evaluation method. Even in these cases, however, authors called for prospective studies to better understand the tools’ influence on patient decisions and outcomes. Likewise, early-stage CDSS prototypes targeting broader health system planning [13] were recommended for further evaluation of their computational performance and integration of advanced modeling techniques to support strategic decision-making.

### Ethical and regulatory considerations

The studies included in this review showed a wide range of approaches to ethical and regulatory compliance. Several studies did not explicitly report ethics approvals or considerations [3, 10, 16], while others clearly stated that their protocols had been reviewed and approved by an Institutional Review Board (IRB) and included appropriate data deidentification measures [13, 17, 18]. Compliance with data privacy regulations such as HIPAA was documented in studies involving patient-level data in the United States [20].

Most U.S.-based studies received IRB approval [13, 18, 19]. For studies conducted in international settings, ethical approval was typically granted by local hospital ethics committees or national regulatory bodies [7, 11, 14]. For instance, the study in Kenya adhered to guidelines from the Ministry of Health [14].

In cases where the research was retrospective or did not involve direct patient interaction, ethics approval was often deemed unnecessary or was only briefly acknowledged [9, 22]. This was particularly common in studies focused on secondary data analysis or non-interventional simulations.

Prospective studies, especially those involving newborns or vulnerable populations, more consistently reported formal ethical procedures, including informed consent and compliance with national registries [15, 24]. Several studies also emphasized the need for future clinical trials, highlighting the importance of ongoing ethical oversight in the development and deployment of CDSS in maternal and neonatal health settings [24].

## Discussion

This scoping review mapped the current landscape of CDSS that integrate interpretable ML models for high-risk pregnancy prevention. The findings reflect a growing interest in using advanced modeling techniques in maternal healthcare, with noticeable progress in predictive accuracy, transparency, and reported clinical outcomes. At the same time, the review uncovered persistent challenges, particularly related to data quality, model generalizability, and the seamless integration of these systems into clinical practice. In this section, we contextualize our results within the broader literature and outline key enablers, limitations, and priorities for future research. Our decision to include both ML-based and non-ML CDSS reflects real-world diversity: many clinical systems rely on rule-based logic or hybrid architectures that share interpretability goals with machine learning models. Including this full spectrum allows a more complete understanding of how transparency and usability are operationalized in maternal decision support.

One of the most notable trends in the reviewed studies is the adoption of interpretability techniques such as SHAP and LIME. These tools aim to increase interpretability of critical factors in high-stakes clinical environments. SHAP, in particular, was commonly used in models predicting preeclampsia and preterm birth, helping clinicians understand which variables contributed most to individual predictions [18, 19]. This aligns with broader efforts in the field to prioritize interpretability as a prerequisite for clinical adoption [25].

Nonetheless, applying interpretability methods to complex architectures like deep neural networks remains a challenge. Some studies employed deep learning models such as CNNs and RNNs to analyze cardiotocograms and fetal heart rate data [19, 24]. Despite their high accuracy, these models are often seen as “black boxes,” limiting their usefulness in clinical decision-making. Techniques like Grad-CAM offer visual explanations but fall short in capturing the dynamic and temporal dependencies critical for maternal-fetal monitoring [26].

Several ML models in the review, particularly Random Forest and Support Vector Machines, achieved excellent discrimination, with reported AUCs exceeding 90% in tasks such as cesarean section classification and preeclampsia risk prediction [3, 18]. These results echo findings from other studies showing ML’s advantage over traditional statistical models in maternal health [4].

However, predictive performance alone is insufficient for real-world implementation. Clinical usability, interpretability, and seamless integration into care workflows are equally essential. For instance, although the Cox-nnet model demonstrated strong predictive power [18], it required external validation to ensure its applicability in different settings, highlighting the common issue of overfitting [27]. Meanwhile, the New Jersey Maternal Mortality Dashboard [16] illustrated how even relatively simple tools, when well-integrated, can meaningfully support clinical and policy decisions.

### Data quality and generalizability

Data limitations were a recurring issue, particularly in studies conducted in low- and middle-income countries (LMICs). While studies from high-income countries had access to large, well-structured datasets, those from LMICs frequently encountered small sample sizes, missing data, and class imbalance [12, 23]. Although methods like SMOTE were used to address these imbalances, such techniques can introduce overfitting if not carefully validated. These challenges mirror broader concerns about the uneven distribution of high-quality health data and the risks this poses for equitable AI deployment [28].

Concerns about model generalizability were also well-founded. Several models trained in high-resource environments showed reduced performance when applied to other contexts [18], reaffirming the importance of external validation and adaptation before large-scale deployment [27]. Without careful testing in diverse populations, there is a risk that these tools could reinforce existing healthcare disparities.

Even the most accurate models may fail to make a clinical impact if they are not user-friendly or well-integrated into clinical workflows. Several studies reported difficulties such as alert fatigue, poor user interface design, or workflow misalignment. For example, in the case of the AlertWatch OB system, the high frequency of automated alerts led to reduced clinician responsiveness, an issue echoed across the CDSS literature [4, 22].

Successful implementation depends heavily on clinician engagement during design and testing phases. Studies showed that CDSS tools were more likely to be adopted when they complemented existing clinical routines and provided actionable insights [9, 20]. This underscores the need for co-design approaches and ongoing usability testing as part of CDSS development pipelines [28].

The findings of this review suggest several clear avenues for future research and development. First, real-world studies are needed to evaluate the real-world impact of ML-based CDSS on maternal and neonatal outcomes. Many existing studies stop at performance metrics without assessing downstream clinical effects.

Second, the field must continue to invest in XAI, particularly for complex models. As deep learning models become more common, ensuring that their outputs are interpretable to clinicians, especially those without technical background, will be vital for trust and adoption.

Third, improving data equity should be a priority. Building international collaborations, promoting open-access datasets, and developing context-adapted models are essential steps to support model generalizability and fair deployment across settings.

Finally, embedding human-centered design principles throughout development, from needs assessment to deployment, can help ensure that CDSS are practical, usable, and ultimately impactful in everyday clinical care.

## Conclusion

This review underscores the potential of interpretable ML to enhance clinical decision-making in the context of high-risk pregnancy prevention. While advances have been made in model performance, interpretability, and application, key challenges remain. These include limited generalizability, insufficient real-world validation, and gaps in integration with clinical workflows. Addressing these challenges will require a combination of methodological innovation, ethical data sharing, and clinician-centered design. With these advances, CDSS can become powerful tools in the ongoing effort to improve maternal and neonatal health outcomes.

## Data Availability

Not applicable. This study is based on previously published literature.
